# Progress in Genetic Polymorphisms Related to Lipid Disturbances Induced by Atypical Antipsychotic Drugs

**DOI:** 10.3389/fphar.2019.01669

**Published:** 2020-02-04

**Authors:** Nana Li, Ting Cao, Xiangxin Wu, Mimi Tang, Daxiong Xiang, Hualin Cai

**Affiliations:** ^1^ Department of Pharmacy, The Second Xiangya Hospital of Central South University, Changsha, China; ^2^ Institute of Clinical Pharmacy, Central South University, Changsha, China; ^3^ Department of Pharmacy, Xiangya Hospital, Central South University, Changsha, China; ^4^ Institute of Hospital Pharmacy, Xiangya Hospital, Central South University, Changsha, China

**Keywords:** atypical antipsychotic drugs, weight gain, metabolic syndrome, pharmacogenomics, single nucleotide polymorphisms, leptin, 5-HT_2C_ receptor

## Abstract

Metabolic side effects such as weight gain and disturbed lipid metabolism are often observed in the treatment of atypical antipsychotic drugs (AAPDs), which contribute to an excessive prevalence of metabolic syndrome among schizophrenic patients. Great individual differences are observed but the underlying mechanisms are still uncertain. Research on pharmacogenomics indicates that gene polymorphisms involved in the pathways controlling food intake and lipid metabolism may play a significant role. In this review, relevant genes (*HTR2C*, *DRD2*, *LEP*, *NPY*, *MC4R*, *BDNF*, *MC4R*, *CNR1*, *INSIG2*, *ADRA2A*) and genetic polymorphisms related to metabolic side effects of AAPDs especially dyslipidemia were summarized. Apart from clinical studies, *in vitro* and *in vivo* evidence is also analyzed to support related theories. The association of central and peripheral mechanisms is emphasized, enabling the possibility of using peripheral gene expression to predict the central status. Novel methodological development of pharmacogenomics is in urgent need, so as to provide references for individualized medication and further to shed some light on the mechanisms underlying AAPD-induced lipid disturbances.

## Introduction

Schizophrenia is a severe mental disorder with a lifetime morbid risk of approximately 1% across the world (McGrath et al., 2008). Continuous treatment with sufficient dosage of antipsychotic drugs is essential in the therapy and management of schizophrenia (Emsley, 2018). Second-generation antipsychotics [also called atypical antipsychotic drugs (AAPDs)] are first-line antipsychotics with greater improvement of negative symptoms and fewer extrapyramidal symptoms than first-generation antipsychotics. However, metabolic side effects (e.g., weight gain, dyslipidemia, hyperglycemia, etc.) induced by AAPDs raise the risk of cardiovascular diseases, which results in patient noncompliance, relapse, and increased mortality (Mottillo et al., 2010; Mitchell et al., 2013; Ringen et al., 2014). Several studies have reported that antipsychotic-induced weight gain is reversible among pediatric and adult patients who discontinued treatment of antipsychotics (de Kuijper et al., 2013; Upadhyay et al., 2019). It is usually uneasy to make an optimum choice since benefits of these drugs have to be weighed against risks.

Although there have been tremendous reports on the metabolic side effects of atypical antipsychotic drugs, the mechanisms remain elusive (Reynolds and McGowan, 2017). Available evidence has suggested that the clinical responses to antipsychotics and related side effects could vary from patient to patient. The large variability can be attributed to a variety of complex factors, in which genetic factors may play a dominant role. Numerous studies on pharmacogenomics have been conducted to elucidate gene variants related to antipsychotic-induced weight gain or metabolic disturbances (Lett et al., 2011; Zhang et al., 2016; Zai et al., 2018). A meta-analysis has revealed that the genes of pharmacodynamic targets of antipsychotics like *HTR2C*, *DRD2*, *ADRA2A* and genes implicated in obesity such as *MC4R*, *GNB3*, *FTO*, *LEP*, *LEPR*, *BDNF*, and *INSIG2* seem to be consistently relevant to antipsychotic-induced weight gain (Zhang et al., 2016). The current systematic review aims to provide an update on the gene polymorphisms related to lipid disturbances of AAPDs and to find the possible relations between central and peripheral pathways.

## Methods

**Figure 1:**
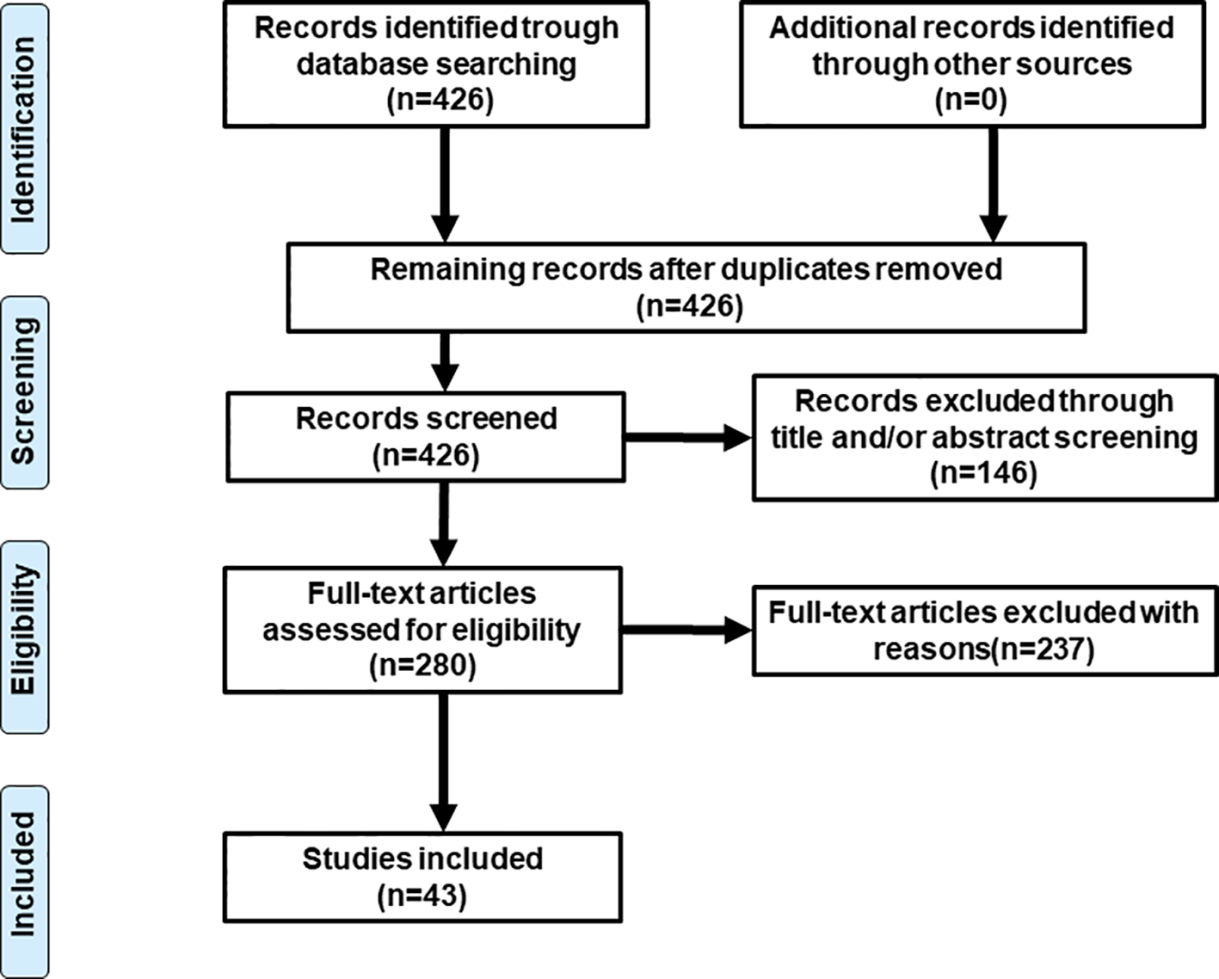
Flowchart of study selection.

Literature research was conducted on PubMed (last: 31 October 2019) with the combinations of the key words: antipsychotic* neuroleptic*, gene, pharmacogen*, polymorphism, weight gain, metabolic, and dyslipidemia. Inclusion criteria were: 1) patients with mental illness; 2) under the treatment of atypical antipsychotic drugs; 3) specific gene polymorphisms were studied; 4) outcomes involved in lipid metabolism such weight, BMI, and percentage of metabolic syndrome, etc. Exclusion criteria were: 1) a review or letter; 2) studies on animals; 3) studies of genes not examined in other studies. Totally, 43 studies were selected for this review (see **Figure 1** and **Table 1** for details).

**Table 1:** Demographic, treatment, and lipid parameters of mentioned studies.

Reference	Genes (SNPs)	Functional SNPs	Risk allele	Period	APs (n)	% Caucasian	Sample size (M/F), %FEDN	Age	Diagnoses	Single/multi-center	Lipid parameters
Reynolds et al., 2002	*HTR2C* rs3813929 (-759C/T)	Yes	C	6, 10 wk	CP (69), RIS (46), CLO (4), FLU (3), SUL (1)	0 (100% Asian)	123 (61/62), 100%	26.6 ± 7.7	SCZ	Single	BMI change, >7% weight gain
Templeman et al., 2005	*HTR2C* rs3813929	–	C	6 wk, 3 mo, 9 mo	RIS (26), OLZ (19), HAL (10), QUE (11), ZIP (6), AMI (1)	100	73 (55/18), 100%	25.2 ± 0.78	Psychosis	Single	BMI change, >7% BMI increase
	*LEP* rs7799039 (-2548A/G)	Yes	G								
Ryu et al., 2007	*HTR2C* rs3813929	–	C	4 wk	RIS (53), OLZ (12), AMI (5), QUE (4), SGAs (10)	0 (100% Asian)	84 (39/45), 69%	30.1 ± 7.4	SCZ	Single	>5, >7% BMI increase
Mulder et al., 2007	*HTR2C* rs3813929	–	NS	Cross-sectional study	CLO, OLZ, RIS (> 80%); others	88	112 (74/38), 0%	36 ± 10	SCZ (63%), SZA (25%), others (12%)	Single	Presence of MS, HDL-C, TG, Waist, BP
	*HTR2C* rs518147 (-697G/C)	Yes	C								
Kuzman et al., 2008	*HTR2C* rs3813929	–	NS	4 mo	OLZ (61), RIS (47)	100	108 (0/108), 89%	30.6 ± 11.5	SCZ, SZA	Two sites	≥7% weight gain
Sicard et al., 2010	*HTR2C* rs3813929	–	NS	6 wk	CLO or OLZ (130), others	68	205 (141/64), N/A	35.9 ± 10.1	SCZ, SZA	Multiple	>7% weight gain, % weight change
	*HTR2C* rs518147	–	G								
	*HTR2C* rs6318 (Ser23Cys)	Yes	NS								
Kuzman et al., 2011	*HTR2C* rs3813929	–	T	3 mo	OLZ (61), RIS (40)	100	101 (0/101), 100%	33.47 ± 10.62	SCZ (76%), SZA (7%), delusional disorder (17%)	Single	FBG, TC, LDL, HDL, TG, BP, Waist, Hip; occurrence of MS
Hong et al., 2010	*DRD2* rs4436578	N/A	C	48.2 ± 27.8 mo	CLO (239), OLZ (70), RIS (170)	0 (100% Asian)	479 (292/187), 0%	47.2 ± 13.2	SCZ	Single	≥7% weight gain
Lencz et al., 2010	*DRD2* rs1799732 (-141C Ins/Del)	Yes	Del	6 wk	RIS (32), OLZ (26)	28 (40% African-American)	58 (44/14), 100%	23.5 ± 4.9	SCZ	Two sites	The log of the ratio of weight relative to baseline weight
Muller et al., 2012	*DRD2* rs6277 (-957C/T)	N/A	C	6, 14 wk	CLO or OLZ (132), others	62 (28% African American)	206 (141/65), N/A	35.69 ± 19.55	SCZ, SZA	Three sites	≥7% weight gain, % weight change
	*DRD2* rs1079598	N/A	C								
	*DRD2* rs1800497 (TaqIA)	N/A	A1 (T)								
Tybura et al., 2014	*DRD2* rs1799732	–	NS	12 wk	ZIP (59), OLZ (82), perazine (60)	100	191 (89/102), N/A	36.1 ± 12.4	SCZ	Two sites	Average weight change
	*DRD2* rs1800497	–	NS								
Alladi et al., 2017	*DRD2* rs1799732	–	NS	4 wk	RIS	100% South Indian	289 (175/114), N/A	35.3 ± 10.0	SCZ	Single	>5% weight gain
	*HTR2C* rs3813929	–	NS								
de Leon et al., 2008	*NPY* rs1468271	N/A	G	Cross-sectional study	OLZ, QUE, or CP (165); others (192)	88	357 (229/128), 0%	37.6 ± 10.6	Severe mental illnesses	Three sites	Serum glucose, TC, HDL-C, TG
Tiwari et al., 2013	*NPY* rs10551063	N/A	NS	7.03 ± 3.4 wk	CLO (99), HAL (18), OLZ (37), RIS (40), others (32)	70 (25% African American)	226 (151/75), 58%	35.78 ± 10.6	SCZ, SZA	Three sites	≥7% weight gain, % weight change
	*NPY* rs16147	Yes	C								
	*NPY* rs5573	Yes	A								
	*NPY* rs5574	Yes	T								
	*NPY* rs16475	Yes	NS								
Malhotra et al., 2012	*MC4R* rs489693	N/A	A	12 wk	RIS (84), QUE (25), ARI (30)	55.4 (23.0% African American)	139 (81/58), 100%	13.38 ± 3.75	Various diagnoses (5% SCZ)	Multiple	BMI change, weight change, fat mass, TG, TC, HDL-C, LDL-C, glucose, insulin, HOMA-IR index, leptin
				6 wk	CLO	70	73 (45/28), 0%	33.48 ± 8.33	SCZ		
				6 wk	RIS (20), QUE (5), ARI (15)	100	40 (22/18), 0%	35.20 ± 11.33	SCZ, SZA		
				12 wk	HAL (31), AMI (21), QUE (25), ZIP (15)	100	92 (53/39), 100%	26.02 ± 5.17	SCZ, SZA, SZP		
Czerwensky et al., 2013a	*MC4R* rs17782313	N/A	C	4 wk	OLZ (135), CLO, RIS, PAL, QUE, AMI	100	345 (138/207), 26%	40.1 ± 14.7	Various diagnoses	Single	% weight gain, % BMI increase
Czerwensky et al., 2013b	*MC4R* rs489693	–	A	4 wk	OLZ (135), CLO, RIS, PAL, QUE, AMI	100	341 (143/198), 25%	41.3 ± 15.0	Various diagnoses	Single	% weight gain, % BMI increase
Chowdhury et al., 2013	*MC4R* rs17782313	–	NS	6-14 wk	CLO (99), HAL (16), OLZ (36), RIS (40), others (33)	70 (25% African American)	224 (150/74), N/A	35.63 ± 10.50	SCZ, SZA	Three sites	% weight gain
	*MC4R* rs11872992	N/A	NS								
	*MC4R* rs8087522	N/A	A								
Zhang et al., 2019	*MC4R* rs489693	–	A	6 wk	ZIP (330), ARI (313), OLZ (341), QUE (332), RIS (344), HAL (166), perphenazine (165)	0 (100% Asian)	1991 (999/992), 28%	31.95 ± 7.93	SCZ	Multiple	>7% BMI gain, % change of BMI, Waist, glucose, TG, HDL, LDL
	*MC4R* rs17782313	–	NS								
Zhang et al., 2008	*BDNF* rs6265 (Val66Met)	Yes	Met	18 ± 6 y	CLO (98), RIS (36), FGAs (62)	0 (100% Asian)	196 (130/66) patients, 0%	N/A	SCZ	Single	BMI gain
							50 (34/16) controls	N/A			
Tsai et al., 2011	*BDNF* rs6265	–	NS	49.2 ± 28.2 mo	CLO (266), OLZ (79), RIS (136)	0 (100% Asian)	481 (293/188), 0%	43.9 ± 8.9	SCZ	Single	% weight gain
	*BDNF* rs11030101	N/A	T								
	*BDNF* rs12291186	N/A	NS								
Zai et al., 2012	*BDNF* rs6265	–	NS	6 wk	CLO or OLZ (207); others	100	257 (188/69), 0%	31.8 ± 7.9	SCZ, SZA	Multiple	≥7% weight gain
	*BDNF* rs1519480	N/A	A								
Zhang et al., 2013	*BDNF* rs6265	–	Met	Cross-sectional study	CLO	0 (100% Asian)	199 (143/56), 0%	55.3 ± 6.9	SCZ	Single	Presence of MS, individual parameters
Fonseka et al., 2015	*BDNF* rs6265	–	Val	7.26 ± 3.5	CLO (87), OLZ (32), RIS (31), HAL (15), others	68 (27% African American)	188 (126/62), 0%	35.8 ± 10.0	SCZ, SZA	Three sites	≥7% weight gain, % weight change
Fang et al., 2016	*BDNF* rs6265	–	Met	Cross-sectional study	CLO (156), RIS (86), SGAs (66)	0 (100% Asian)	308 (205/103) patients, 0%	44.6 ± 10.3	SCZ	Single	BMI
							304 (180/124) controls	44.5 ± 14.4			
Tiwari et al., 2010a	*CNR1* rs806378	N/A	T	7.54 ± 3.55 wk	CLO (101), OLZ (34), HAL (12), RIS (36)	64 (30% African-American)	183 (124/59), 0%	36.12 ± 10.17	SCZ, SCA	Three sites	% weight gain
Monteleone et al., 2010	*CNR1* rs1049353 (-1359G/A)	N/A	NS	24 wk	CLO (25), OLZ (22), RIS (14), QUE (6), ARI (6), HAL (10)	100	83 (50/33) patients, 0%	44.0 ± 10.5	SCZ (86%)	Two sites	>7% weight gain
							80 (40/40) controls	49.4 ± 12.9			
Park et al., 2011b	*CNR1* rs1049353	–	NS	Over 1 y	OLZ	0 (100% Asian)	78 (52/26), N/A	46.4 ± 11.6	SCZ	Three sites	≥7% weight gain
	*CNR1* rs806368	–	NS								
	*CNR1* rs4707436	N/A	NS								
Yu et al., 2013	*CNR1* rs6928499	N/A	G	Cross-sectional study	CLO or OLZ (197); others	95.1	407 (274/133), 0%	35.6 ± 11.0	SCZ (69.3%), SZA (20.1%), SZP (10.6%)	Single	Presence of MS, individual parameters
	*CNR1* rs1535255	N/A	T								
	*CNR1* rs2023239	N/A	T								
Kang et al., 2008	*LEP* rs7799039 (-2548A/G)	Yes	A	453 ± 289 d	OLZ	0 (100% Asian)	74 (50/24), N/A	47.2 ± 11.6	SCZ	Three sites	≥7% weight gain
Yevtushenko et al., 2008	*LEP* rs7799039	–	G	Cross-sectional study	CLO (21), OLZ (31), RIS (16)	100	134 (87/47), 0%	41.6 ± 11.8	SCZ, SZA	Two sites	Presence of MS, Waist, BMI, BMI ≥30 kg/m^2^, presence of central obesity
	*HTR2C* rs3813929 (-759C/T)	–	NS								
Gregoor et al., 2010	*LEP* rs7799039	–	A	Cross-sectional study	OLZ (99), CLO (86), QUE (56), RIS (46), ARI (14)	100	353 (184/169), 0%	N/A	Psychotic disorder (50.7%)	Single	Serum TC/HDL
Gregoor et al., 2011	*LEP* rs7799039	–	NS	within 1 y	CLO (68), OLZ (54), QUE (31), ARI (23), RIS (30)	100	141 (82/59), 0%	N/A	Psychotic disorder (63.1%)	Single	BMI >30, BMI change, BMI change per week
Brandl et al., 2012	*LEP* rs7799039	–	NS	7.19 ± 3.47 wk	CLO (80), OLZ (28), RIS (32), HAL (16), others (24)	70.2 (23.8% African-American)	181 (118/63), N/A	35.93 ± 10.94	SCZ, SZA	Three sites	% weight change
	*LEP* rs10954173	N/A	NS								
	*LEP* rs3828942	N/A	NS								
Le Hellard et al., 2009	*INSIG2* rs17587100	N/A	N/A	12 ± 1.2 wk	CLO	100	160 (97/63), 0%	21.9 ± 8.9	Schizophrenia spectrum disorders	Single	BMI change
	*INSIG2* rs10490624	N/A	N/A								
	*INSIG2* rs17047764	N/A	N/A								
Opgen-Rhein et al., 2010	*INSIG2* rs17587100	–	NS	6 wk	CLO, OLZ, RIS, AMI, QUE, SGAs	100	128 (48/80), 17%	38.63 ± 11.96	SCZ, SZA	Three sites	≥7% weight gain, % weight change
	*INSIG2* rs10490624	–	NS								
	*INSIG2* rs17047764	–	NS								
Tiwari et al., 2010b	*INSIG2* rs17587100	–	NS	7.94 ± 3.74 wk	CLO (96), OLZ (34), RIS (12), HAL (12)	57.8 (35.1% African-American)	154 (110/44), 0%	35.78 ± 9.82	SCZ, SZA	Three sites	≥7% weight gain, % weight change
	*INSIG2* rs10490624	–	NS								
	*INSIG2* rs17047764	–	NS								
Liou et al., 2012	*INSIG2* rs11123469	N/A	C	Cross-sectional study	CLO (171), OLZ (91), RIS (194)	0 (100% Asian)	456 (369/157), 0%	N/A	SCZ	Single	Prevalence of MS
	*INSIG2* rs10185316	N/A	NS								
	*INSIG2* rs1559509	N/A	NS								
Wang et al., 2005	*ADRA2A* rs1800544 (-1291C/G)		G	14.0 ± 6.2 mo	CLO	0 (100% Asian)	93 (49/44), 0%	38.4 ± 8.1	SCZ	Single	Weight gain, >7% weight gain
Park et al., 2006	*ADRA2A* rs1800544	–	G	Over 1 y	OLZ	0 (100% Asian)	62 (44/18), 0%	46.5 ± 11.1	SCZ	Two sites	>10% weight gain, % weight change
Sickert et al., 2009	*ADRA2A* rs1800544	–	C	8.4 ± 3.6 wk	CLO (85), OLZ (20), RIS (13), HAL (11)	50 (42% African-American)	129 (96/33), 0%	36.5 ± 9.0	SCZ, SZA	Multiple	weight gain, % weight change
Risselada et al., 2010	*ADRA2A* rs1800544	–	NS	Cross-sectional study	OLZ (106), RIS (103), CLO (102), ARI (21), QUE (12), SGAs (69)	94	470 (320/150), 0%	38 ± 10	SCZ (78%), SZA (17%)	Multiple	Presence of MS, HDL, TC, Waist, BP, glucose
De Luca et al., 2011	*ADRA2A* rs1800544	–	NS	8.3 ± 3.7 wk	CLO (91), OLZ (22), HAL (12), RIS (14)	51.8 (40.3% African-American	139 (101/38), N/A	36.2 ± 9.4	SCZ	Three sites	% weight change, weight gain >8%

SCZ, schizophrenia; SZA, schizoaffective disorder; SZP, schizophreniform disorder; FEDN, first-episode drug-naïve patients; SNPs, single nucleotide polymorphisms; N/A, not available; NS, not significant; APs, antipsychotics; FGAs, first-generation antipsychotics; FLU, fluphenazine; HAL, haloperidol; CP, chlorpromazine; CLO, clozapine; OLZ, olanzapine; RIS, risperidone; ARI, aripiprazole; QUE, quetiapine; AMI, amisulpride; ZIP, ziprasidone; PAL, paliperidone; SUL, sulpiride; d, days; wk, weeks; mo, months; y, years; M, male; F, female; MS, metabolic syndrome; HDL, high-density lipoprotein; HDL-C, HDL cholesterol; LDL, low-density lipoprotein; LDL-C, LDL cholesterol; FBG, fasting blood glucose; BP, blood pressure; TG, triglycerides; TC, total cholesterol; HOMA-IR index, the homeostasis model assessment insulin resistance index; Waist, waist circumference; Hip, hip circumference.

## Gene Polymorphisms Related to Central Nervous System

### The Serotonin 5-HT_2c_ Receptor

Central serotonin system is associated with the modulation of feeding behavior (Lam et al., 2010). There are at least seven subtypes of 5-HT receptors, of which the 5-HT_2_ subtype is divided into 5-HT_2A_, 5-HT_2B_, and 5-HT_2C_. The serotonin 5-HT_2C_ receptor has shown the most consistent findings in studies on atypical antipsychotic drug-induced lipid disturbances. The serotonin 5-HT_2C_ receptor is present in hypothalamic nuclei such as the arcuate nucleus (ARC) and the ventral tegmental area (VTA) (Faton et al., 2018). Animal experiments have shown that 5-HT_2C_ receptor agonists reduce feeding (Clifton et al., 2000), and the antagonists increase feeding and lead to weight gain (Bonhaus et al., 1997). The serotonin 5-HT_2C_ receptor gene knockout mice ate more than the controls and became obesity (Tecott et al., 1995). Numerous studies have indicated that 5-HT_2C_ receptor mediates leptin-induced anorexia, but reports regarding serotonin-leptin interactions are discrepant (von Meyenburg et al., 2003; Voigt and Fink, 2015; Wierucka-Rybak et al., 2016). Cannabinoid receptor 1 (CB_1_ receptor) stimulation could inhibit the secretion of cerebral serotonin in the mouse brain (Nakazi et al., 2000). Coupled with the inhibition of neuropeptide Y (NPY)/agouti-related protein (AgRP) neurons by 5-HT_1B_ receptor action, 5-HT_2C_ receptor mediates the activation of pro-opiomelanocortin (POMC) neurons as a downstream pathway of serotonin controlling food intake (Lam et al., 2010). Showing the highest antagonizing affinity to 5-HT_2C_ receptor among antipsychotic drugs, clozapine and olanzapine exert the most serious effects of weight gain in patients (Allison et al., 1999). It suggested that the antagonism against 5-HT_2C_ receptor by antipsychotic drugs may lead to increased food intake and eventually weight gain. This is supported by the observation that olanzapine exerts its metabolic side effects by targeting 5-HT_2C_ receptor in mouse model (Lord et al., 2017).

The promoter region of *HTR2C* gene is mainly influenced by -759C/T (rs3813929) and -697G/C (rs518147) polymorphisms. *HTR2C* -759C/T polymorphism was the first single nucleotide polymorphism (SNP) to be reported as an associated *HTR2C* polymorphism with antipsychotic drug-induced weight gain (Reynolds et al., 2002). Besides, it is the most replicated gene polymorphism related to AAPD-induced weight gain (Zhang et al., 2016). Various clinical studies suggested that *HTR2C* -759C allele was a risk allele of a substantial weight gain (over 7% than baseline) in patients treated with typical or atypical antipsychotic drugs (Reynolds et al., 2002; Templeman et al., 2005; Ryu et al., 2007). A study in first episode drug-naive female patients with schizophrenia showed that -759T is associated with an increase in waist circumference, fasting blood glucose, and blood triglyceride levels (Kuzman et al., 2011). However, associations between *HTR2C* -759C/T polymorphism and weight gain or presence of metabolic syndrome were reported to be nonsignificant in other studies (Mulder et al., 2007; Kuzman et al., 2008; Sicard et al., 2010; Alladi et al., 2017). Despite the negative results, haplotype analyses suggested that *HTR2C* 759C-697G-Cys23 haplotype was associated with the most percentage weight gain induced by various antipsychotics (Sicard et al., 2010).

### Dopamine Receptor D2

Dopamine receptor D2 (DRD2) is the main target of antipsychotic drugs. An animal study found that the availability of striatal D2 receptor in obese rats was lower than that in lean controls (Hamdi et al., 1992). In a human study, the striatal dopamine transporter availability was negatively correlated with body mass index (BMI) in a group of healthy volunteers (Chen et al., 2008). It is postulated that it could be a mechanism of overeating that the neuropeptides regulating homeostatic energy balance also modulate the activity of dopamine neurons and the rewarding circuits underlying food intake (Volkow et al., 2011). Therefore, Blum et al. hypothesized that as dopamine D2 receptor antagonists, antipsychotic drugs cause a hypodopaminergic reward circuitry, leading to excessive food intake and ultimately obesity (Blum et al., 2014). Functional magnetic resonance imaging (fMRI) showed that an increased activity in striatal regions of the reward system was positively correlated with weight gain after 6-week amisulpride treatment (Nielsen et al., 2016).

The role of *DRD2* rs4436578-C in weight gain induced by atypical antipsychotics is verified in 479 chronic schizophrenic patients under long-term treatment of clozapine, olanzapine, or risperidone (Hong et al., 2010). *DRD2* promoter region polymorphism -141C Ins/Del (rs1799732) was also reported to be associated with weight gain in 58 first episode patients treated with randomly-assigned olanzapine or risperidone for 6 weeks (Lencz et al., 2010), whereas nonsignificant associations were found in later studies of larger samples (Tybura et al., 2014; Alladi et al., 2017). A systematic analysis of genetic polymorphisms spanning the five dopamine receptor genes (*DRD1*–*DRD5*) found only *DRD2* rs6277 (C957T), rs1079598, and rs1800497 (TaqIA) to be significantly associated with antipsychotic-induced weight gain in chronic patients with schizophrenia or schizoaffective disorder (Muller et al., 2012). It was suggested that the C957T polymorphism would not change the amino acid sequence of the dopamine D2 receptor, but it was related to the stability of the striatum D2 receptor (Hirvonen et al., 2009) and the stability and half-life of the *DRD2* messenger RNA (mRNA) (Duan et al., 2003). The TaqIA polymorphism is located in coding region of the adjacent *ANKK1* gene and overlaps with the 3’ end region of the *DRD2* gene. So, it may be in linkage disequilibrium with a functional polymorphism of *DRD2*, or may affect the dopamine signaling through the *ANKK1* gene. Studies have shown that if the TaqIA site is A1, it may result in decreased expression of *DRD2* and decreased dopaminergic activity (Giegling et al., 2013).

### Neuropeptide Y

NPY is a 36-amino-acid peptide expressed in the central and peripheral nervous system. In the ARC neurons, NPY colocalizes with AgRP, which can antagonize α-melanocyte-stimulating hormones (α-MSH) binding to melanocortin-3 receptor (MC3R) and melanocortin-4 receptor (MC4R). Another group of neurons co-express POMC and cocaine and amphetamine-regulated transcript (CART) and inhibit food intake (Arora and Anubhuti, 2006). Low leptin levels can upregulate neuropeptide Y and exert orexigenic effects. Leptin directly and differentially regulates NPY and POMC neurons, and then controls feeding behavior and energy homeostasis (Elias et al., 1999). Outside the hypothalamus, NPY mainly exists in the brainstem and the catecholaminergic neurons in the sympathetic nervous system. Results *in vitro* indicate that NPY may inhibit lipolysis in murine adipocytes (Bradley et al., 2005). Transgenic mice overexpressing NPY showed significant obesity and the lipogenic effects as well as inhibition of catecholaminergic tone of NPY were suggested (Vahatalo et al., 2015). NPY and leptin are recognized to interact in a homeostatic loop to regulate energy balance not only in the brain, but also directly at the adipocyte level (Martinez et al., 2000).

Chronic treatment of atypical antipsychotic drugs increased NPY immunoreactivity and mRNA expression in the rat hypothalamus (Kirk et al., 2006; Weston-Green et al., 2012). *NPY* rs1468271 was associated with hypercholesterolemia in patients taking olanzapine, quetiapine, or chlorpromazine (de Leon et al., 2008). Significant associations between the SNPs rs16147, rs5573, and rs5574 in *NPY* and weight gain in clozapine or olanzapine-treated patients of European ancestry were reported (Tiwari et al., 2013). Compared with carriers of TT genotype at rs16147, individuals with the C allele showed a higher risk of weight gain probably due to increased NPY levels. Besides, genetic interaction between rs16147 in *NPY* and rs806378 in cannabinoid receptor 1 gene further supports their biological interaction (Tiwari et al., 2013).

### The Melanocortin 4 Receptor

The MC4R is a transmembrane G protein-coupled receptor expressed in the hypothalamus and peripheral tissues. The central melanocortin system potently regulates feeding and directly controls lipid metabolism in liver and adipocytes (Nogueiras et al., 2007). MC4R plays a key role in suppressing food intake. *MC4R* gene is the most common single-gene effect of human obesity (Beckers et al., 2011). Rodent experiments showed that *Mc4r* knockout mouse exhibited hyperinsulinemia, hyperglycemia, and adult obesity syndrome (Srisai et al., 2011). Multiple pathways are involved in the regulation of central melanocortin system in energy homeostasis (Shen et al., 2017). MC4R signaling regulates energy balance through stimulating brain-derived neurotrophic factor (BDNF) expression in the ventromedial hypothalamus (VMH) (Xu et al., 2003). Central melanocortin pathway through MC4R is an indispensable downstream mediator of the anorexigenic effect of serotonin (Lam et al., 2008). The expression of MC4R in the rat hypothalamus is increased under the long-term treatment of antipsychotics probably through a compensatory mechanism (Rojczyk et al., 2015). A genome-wide association study found that *MC4R* rs489693 demonstrated consistent effects on weight gain, as well as on levels of triglycerides, leptin, and insulin, HOMA-IR index (the homeostasis model assessment insulin resistance index), and total fat mass (Malhotra et al., 2012). Afterward, the association between *MC4R* rs489693 A-allele and greater weight gain was confirmed (Czerwensky et al., 2013b). They also reported carriers of *MC4R* rs17782313 C-allele at risk of greater percentage weight gain after taking atypical antipsychotics (Czerwensky et al., 2013a). However, Chowdhury et al. didn’t replicate the significant association of rs17782313, but they reported that carriers of rs8087522-A gained significantly more weight than non-carriers in white Americans (Chowdhury et al., 2013). Located in the *MC4R* promoter region, rs8087522 A-allele may affect the gene expression of *MC4R* by binding to an unknown nuclear protein, while the G-allele has no effect. A large-scale pharmacogenetic study in Chinese schizophrenia patients reported the ubiquitous association between rs489693 and metabolic measures, while rs17782313 is less involved in antipsychotic-induced metabolic disturbances (Zhang et al., 2019).

### Brain-Derived Neurotrophic Factor

The BDNF is a member of the neurotrophic factor family abundantly expressed in the hippocampus and hypothalamus. Beyond a fundamental role in the brain development and plasticity, BDNF is thought to play a major part in the regulation of food intake (Rosas-Vargas et al., 2011). It is reported that central infusion of BDNF can induce dose-dependent food restriction and weight loss, perhaps *via* its up-regulation of hypothalamic serotonin activity (Pelleymounter et al., 1995). BDNF was also observed to regulate food intake *via* its inhibitory effect on NPY and modulation of the dopamine system (Wang et al., 2007; Cordeira et al., 2010). Researchers have found reduced serum BDNF levels in first-episode drug naive psychosis patients and a trend of greater reductions in female patients (Jindal et al., 2010). Moreover, a significant increase in BDNF levels in prefrontal cortex and cerebrospinal fluid samples of postmortem schizophrenia patients was reported (Issa et al., 2010). Thus, alterations in BDNF may play a role in the pathophysiology of schizophrenia (Favalli et al., 2012). Both typical (haloperidol) and atypical antipsychotic drugs (clozapine, risperidone) decrease serum BDNF levels in schizophrenia patients (Xiu et al., 2009) and the expression of *Bdnf* mRNA in the hippocampus of rats (Angelucci et al., 2000; Lipska et al., 2001; Chlan-Fourney et al., 2002) although results are inconsistent in some studies (Bai et al., 2003; Park et al., 2011a). Further, reduced serum BDNF levels may be related with weight gain in female but not in male patients with schizophrenia under long-term antipsychotic treatment (Zhang et al., 2007). The effect of BDNF on weight gain induced by antipsychotics seems to be gender-specific but the results are inconsistent.

Human *BDNF* gene is located on chromosome 11p14.1. The Val66Met variant (rs6265) in the *BDNF* promoter region is the most investigated SNP of the gene, showing an association of cognitive impairment (Bath and Lee, 2006) and obesity (Skledar et al., 2012). It markedly alters the intracellular trafficking and packaging of pro-BDNF and impacts the activity-dependent secretion of the mature peptide (Egan et al., 2003). The Met/Met genotype of the *BDNF* Val66Met polymorphism has a significant effect on BMI gain and metabolic syndrome in male but not female schizophrenic patients treated with long-term antipsychotic drugs (Zhang et al., 2008; Zhang et al., 2013; Fang et al., 2016). However, these results are incompatible with some studies that indicate Val/Val was associated with greater weight gain induced by antipsychotics (Fonseka et al., 2015). Tsai et al. failed to replicate the relationship between the Val66Met polymorphism and body weight gain after long-term antipsychotic treatment, but they found a visible difference in percentage weight change in patients with different copies of haplotype GTA (rs6265-rs11030101-rs12291186) (Tsai et al., 2011). Additionally, a two-marker haplotype rs6265-rs1519480 was also reported to be associated with antipsychotic-induced weight change in European ancestry (Zai et al., 2012).

### The Cannabinoid 1 Receptor

The endocannabinoid system is involved in modulating energy homeostasis by controlling food intake *via* central and peripheral pathways, as well as stimulating lipogenesis and fat accumulation (Di Marzo and Matias, 2005; Bluher et al., 2006). It may be negatively regulated by leptin in the neural circuitry (Di Marzo et al., 2001). In the hypothalamus, the interaction between the endocannabinoid and NPY systems appears to be bidirectional, and peripheral endocannabinoid levels are increased in obese mice induced by neuropeptide Y overexpression (Vähätalo et al., 2015). Encoding by the gene *CNR1*, the CB_1_ receptor mediates the effects of cannabinoid binding primarily in the brain and also presents in peripheral tissues, including adipocytes (Bensaid et al., 2003), hepatocytes (Osei-Hyiaman et al., 2005), pancreas (Nakata and Yada, 2008), muscle (Mendizabal-Zubiaga et al., 2016), and the gut (Coutts and Izzo, 2004). *Cnr1* knockout mice experienced food restriction compared with wild-type littermates (Di Marzo et al., 2001). Selective CB_1_ receptor antagonist SR141716A (rimonabant) ameliorates diet-induced obesity of mice through enhancement of fatty acid oxidation and energy expenditure in white adipocytes (Jbilo et al., 2005), or modulating macrophage inflammatory mediators *via* gut microbiota alterations (Mehrpouya-Bahrami et al., 2017). Clinical trials proved that rimonabant decreased body weight and waist circumference in overweight or obese patients (Van Gaal et al., 2005; Pi-Sunyer et al., 2006). The 3813G allele at the exon 4 of *CNR1* is associated with obesity-related phenotypes like waist circumference and subscapular skinfold thickness in adult men (Russo et al., 2007).

Evidence from pre-clinical, clinical, genetic, postmortem, and neuroimaging studies have indicated an important role of the endocannabinoid system and cannabinoid receptors in the pathophysiology of schizophrenia (Fakhoury, 2017). The G allele frequency of the *CNR1* 1359G/A gene polymorphism potentially relates to therapeutic response to atypical antipsychotics (Hamdani et al., 2008). Alterations in CB_1_ receptor-mediated G-protein signaling by antipsychotic treatment was different in a sex- and age-selective manner (Wiley et al., 2008). Chronic treatment with aripiprazole upregulated the gene expression of *Cnr1* in the frontal cortex of rats (Cheng et al., 2008). Risperidone increased CB_1_ receptor binding in rat brain (Secher et al., 2010). Oral intake of haloperidol or olanzapine produces region-specific increase in cannabinoid receptor levels distinctly (Delis et al., 2017). Both CB_1_ receptor antagonist NESS06SM and inverse agonist rimonabant reduced food intake and weight gain and restored all blood parameters in a rat model treated with olanzapine (Lazzari et al., 2017). However, the results of the relationship between *CNR1* polymorphisms and antipsychotic-induced lipid disturbances differ from various single nucleotide polymorphisms. A study of 20 tag SNPs found the rs806378 polymorphism to be associated with weight gain in European patients treated with clozapine or olanzapine (Tiwari et al., 2010a). *CNR1* polymorphisms -1359 G/A (rs1049353, rs806368, and rs4707436) were not associated with antipsychotic-induced weight gain (Monteleone et al., 2010; Park et al., 2011b). A cross-sectional study of a naturalistic cohort of 407 patients with schizophrenia showed the minor alleles of rs6928499, rs1535255, and rs2023239 were associated with lower levels of high-density lipoprotein cholesterol and fasting glucose (Yu et al., 2013).

## Gene Polymorphisms Related to Peripheral Tissues

### Leptin

Leptin is a peptide hormone predominantly secreted by adipocytes, targeting hypothalamic nerve network to suppress appetite. At peripheral level, leptin is involved in the regulation of lipid and glucose metabolism in adipose tissue, liver, and skeletal muscle, as well as gastrointestinal nutrient absorption (Sáinz et al., 2015). Leptin resistance primarily takes responsibility for obesity in some studies (Sáinz et al., 2015). Serum leptin levels were elevated significantly after treatment of olanzapine, clozapine, and quetiapine, whereas haloperidol and risperidone produced nonsignificant leptin changes (Potvin et al., 2015). The significant association between leptin increases and BMI changes was observed across studies. Two hypotheses about the role played by leptin in antipsychotic-induced weight gain were proposed: leptin as an epiphenomenon of weight gain, or antipsychotic-induced leptin resistance causing weight gain (Panariello et al., 2012).

Therefore, the correlation between leptin gene (*LEP*) and lipid disturbances induced by atypical antipsychotics has been a research hotspot. Among the polymorphisms, *LEP* rs7799039 (-2548A/G) was verified to be associated with weight gain in many studies (Templeman et al., 2005; Kang et al., 2008; Shen et al., 2014). A cross-sectional study showed that serum total cholesterol (TC)/high-density lipoprotein (HDL) ratio in *LEP* -2548G male carriers was lower than that of non-carriers after taking AAPDs for more than 3 months, but not significant in female patients (Gregoor et al., 2010). However, a longitudinal study conducted by the same group found *LEP* -2548G was not significantly associated with BMI change during treatment of atypical antipsychotics (Gregoor et al., 2011). A haplotype of *LEP* rs7799039G-rs10954173G-rs3828942G showed a significant association with weight gain despite results of all the SNPs were not significant (Brandl et al., 2012). A meta-analysis indicated that the *LEP* -2548A allele was associated with an increased risk of antipsychotic-induced weight gain in Asian patients, while it seemed to decrease the risk in European populations (Shen et al., 2014). Combined genotype analysis revealed that gene-gene interaction between the *LEP* and *HTR2C* polymorphisms was highly significant in their associations with occurrences of metabolic syndrome, BMI, and waist circumference (Yevtushenko et al., 2008).

### Insulin-Induced Gene 2

In the endoplasmic reticulum (ER), insulin-induced gene (INSIG) proteins form complexes with sterol-regulatory element-binding proteins (SREBPs) and SREBP cleavage activating proteins (SCAP), regulating cholesterol and lipid fatty acid biosynthesis (McPherson and Gauthier, 2004). There are two isoforms of INSIG proteins, INSIG1 and INSIG2. INSIG2 is not a transcriptional target of SREBPs as INSIG1, but it can also cause the retention of the SCAP/SREBP complex in the ER in a sterol dependent way and thereby blocks cholesterol synthesis (Yabe et al., 2002). The INSIG2/SCAP/SREBP signaling may be altered by various antipsychotic drugs. Both clozapine and haloperidol can activate the gene expressions of the SREBP system in human glioma cells, which may be a mechanism of therapeutic efficacy (Ferno et al., 2005). But the upregulation of the lipogenesis in peripheral tissues can be a cause of the metabolic side effects induced by antipsychotics. Clozapine and risperidone significantly reduced INSIG2 and activated the expression of SCAP/SREBP in rat liver (Cai et al., 2015). It is reported that AAPD treatment induces early-stage lipid biosynthesis in adipose-derived stem cells (ASCs) and such abnormal lipogenesis can be reversed when INSIG2 expression was increased (Chen et al., 2017). Three markers (rs17587100, rs10490624, and rs17047764) localized within or near the *INSIG2* gene had a strong association with clozapine-induced BMI gain in German patients with schizophrenia (Le Hellard et al., 2009). However, significant associations of the three aforementioned SNPs weren’t replicated in other European patients (Opgen-Rhein et al., 2010; Tiwari et al., 2010b). Liou et al. demonstrated that the C-C-C haplotype of *INSIG2* rs11123469-rs10185316-rs1559509 significantly elevated the risk of AAPD-induced metabolic syndrome (Liou et al., 2012). This association can be attributed to the action of *INSIG2* independently or the gene-gene interaction with *INSIG1*.

### Adrenergic Alpha-2a Receptor

The sympathetic nervous system regulated by the hypothalamus plays an important role in energy expenditure and lipolysis. Adrenergic α-2 receptors are classified into three subtypes, α_2A_, α_2B_, and α_2C_. Mice lacking α_2A_-adrenoceptors (ADRA2A) showed increased energy expenditure, lipolysis, and hyperinsulinemia (Ruohonen et al., 2018). Atypical antipsychotics have affinities for adrenergic receptors, including subtypes of α_2A_, α_2B_, α_2C_, α_1A_, α_1B_ (Roth et al., 2004). The -1291 C/G promoter polymorphism (rs1800544) located in the regulatory promoter sequence of the *ADRA2A* gene may influence the transcription factor control. Association between *ADRA2A* rs1800544 polymorphism and schizophrenia was found in a study of Czech male patients with schizophrenia (Lochman et al., 2013). Carriers of *ADRA2A* 1291-GG gained more weight than the subjects with genotype 1291-CC in Asian patients after long-term treatment of clozapine or olanzapine (Wang et al., 2005; Park et al., 2006). But results of European-Americans showed the carriers of the *ADRA2A* -1291C allele gained more weight during treatment of 8.4 weeks on average (Sickert et al., 2009). The association between *ADRA2A* -1291C/G and the prevalence of metabolic syndrome wasn’t significant among white patients using antipsychotics (Risselada et al., 2010). No significant association between *ADRA2A* -1291C/G and weight gain could be detected in another study among complex ethnic subjects treated with different antipsychotic drugs (De Luca et al., 2011). Conflicting results might be attributed to ethnic differences and diverse observation periods.

## Relations Between Genes in Central Nervous System and Peripheral Tissues

**Figure 2:**
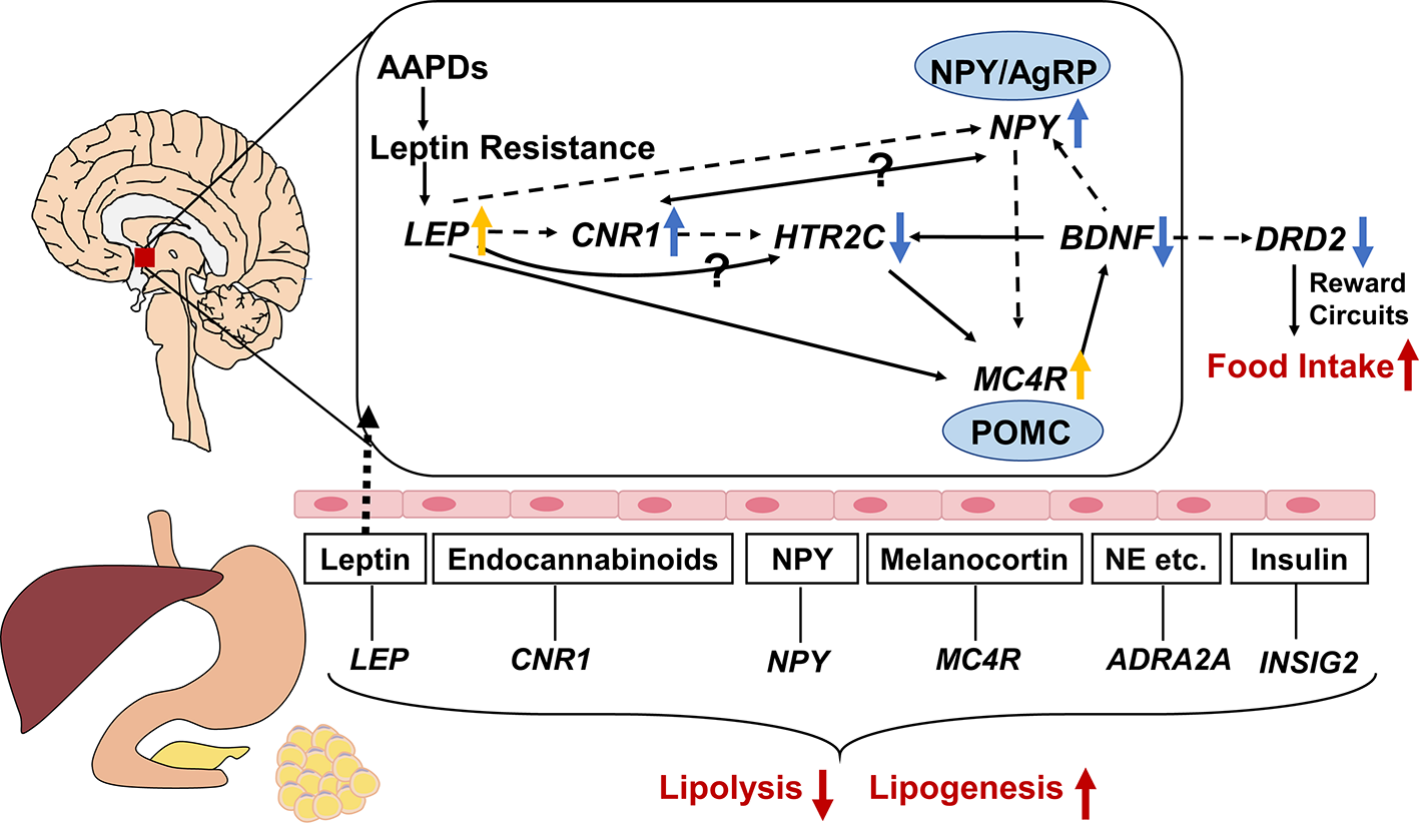
Gene-gene interaction among genes associated with atypical antipsychotic-induced lipid disturbances. The central and peripheral pathways are separated by brain-blood barrier, with leptin through it. Neuropeptide Y (NPY)/agouti-related protein (AgRP) neurons and pro-opiomelanocortin (POMC) neurons play a fundamental role in the downstream of the pathway of *LEP*, *CNR1*, and *HTR2C*. NPY is an orexigenic mediator whereas melanocortin-4 receptor (MC4R) exert anorexigenic effects. *HTR2C* mediates the effects of multiple genes, such as *LEP*, *CNR1*, and *BDNF*. Dopamine regulate appetite mainly through brain reward circuits. As a bridge between the central and peripheral ways, leptin inhibits food intake by triggering multiple signaling cascades like *NPY*, *MC4R*, *CNR1*. In peripheral tissues, multiple hormones or peptides modulating lipid metabolism, such as leptin, neuropeptide Y, melanocortin, endocannabinoids, insulin, and norepinephrine etc. Inhibition of lipolysis and stimulation of lipogenesis lead to hyperlipemia and obesity. The solid and dotted lines indicate upregulation and downregulation respectively. ↑: activated by AAPDs; ↓: inhibited by AAPDs; yellow arrows indicate compensatory effects rather than direct effects; NE, norepinephrine.

Gene expression in central nervous system is not readily available, therefore, it is of great importance to find equivalent evidence from the peripheral blood. It was reported that gene expression in peripheral blood mononuclear cells could be used as a fingerprint of central nervous system disease (Achiron and Gurevich, 2006). Thus, we focus on the network connections between the regulatory mechanisms of central nervous system pathways and peripheral pathways. The connection of the overall related genes mentioned in this review is shown in **Figure 2**. It is obvious that antipsychotics might induce weight gain or metabolic syndrome through central and peripheral ways. In central nervous system, *HTR2C*, *DRD2*, *LEP*, *NPY*, *MC4R*, *BDNF*, *CNR1* polymorphisms play an important role in regulating food intake, and they can be affected by AAPDs. Besides, the lipid metabolism in peripheral tissues may be altered by the SNPs of *LEP*, *NPY*, *MC4R*, *CNR1*, *INSIG2*, and *ADRA2A*. As we can see in **Figure 2**, complex pathways are involved in the modulation of energy intake and energy expenditure, that is orexigenic and anorexigenic mechanisms. NPY/AgRP neurons and POMC neurons play a fundamental role in the downstream of the pathway of *LEP*, *CNR1*, and *HTR2C*. *NPY* is an orexigenic mediator whereas *MC4R* exerts anorexigenic effects. *HTR2C* mediates the effects of multiple genes, such as *LEP*, *CNR1*, and *BDNF*. Dopamine regulates appetite mainly through brain reward circuits. Leptin is an upstream molecular bridging the peripheral tissue and central nervous system. Secreted from fat cells, leptin acts on the hypothalamus through the blood-brain barrier, and ultimately suppresses appetite by triggering multiple signaling cascades like *NPY*, *MC4R*, *CNR1*. In peripheral tissues, multiple hormones or peptides affect lipid metabolism, such as leptin, neuropeptide Y, melanocortin, endocannabinoids, insulin, and norepinephrine, etc. Inhibition of lipolysis and stimulation of lipogenesis lead to hyperlipemia and obesity.

## Conclusion

Pharmacogenomics helps to find possible genetic polymorphisms related to lipid disturbances induced by atypical antipsychotic drugs and the variation among different patients. Nevertheless, inconsistency and limitations have impeded the progress in this field. As mentioned above, discrepancies often occur between different studies on the same SNP. Several reasons are to be noted. A small sample size may reduce the statistical power; and the representativeness of the sample can be weakened if the frequency of a certain base is low in a small sample. Ethnic, gender, and age differences should be taken into account. Evidence has shown worse lipid metabolic dysfunction in female schizophrenia patients due to antipsychotics (Castellani et al., 2019). Sex differences were also found in gene expression associated with antipsychotic induced weight gain (Sainz et al., 2019). Younger age has been reported to be a risk of greater antipsychotic-related weight gain (Maayan and Correll, 2010; Greil et al., 2013). The degree of the metabolic side effects varies from antipsychotics and doses. Evidence suggested a dose-dependent effect between serum levels and metabolic side effects of clozapine and olanzapine although the relationship between daily dose and metabolic disturbances is not clear. (Simon et al., 2009). First episode drug-naive patients are more sensitive to the AAPDs than chronic ones, which may cause the distinct exposure to the adverse drug reaction. Long-term treatment and short-term treatment might be different in the effects of weight gain or metabolic syndrome. To observe the alteration of BMI, a long enough study duration makes it easier to get a significant result. For instance, *LEP* -2548A/G polymorphism showed nonsignificant association with short-term (6-week and 3-month) weight change but was associated with 9-month antipsychotic-induced weight gain (Templeman et al., 2005). Meanwhile, although metabolic syndrome was verified to be related to weight gain for patients under the treatment of clozapine (Bai et al., 2011), different indicators (BMI or blood lipid levels, etc.) and diverse definitions can lead to inconsistency. Significant end points vary from body weight (or BMI) increase ≥7% of baseline to change of metabolic parameters or presence of metabolic syndrome. Hyperlipidemia (hypertriglyceridemia or hypercholesterolemia) may be the outcome of weight gain or a direct effect of antipsychotics. A rodent experiment found that only olanzapine significantly induced weight increase in rats, but both olanzapine and clozapine elevated blood lipid levels after 9-week treatment at clinic equivalent doses (Liu et al., 2017). Therefore, different outcome variables of lipid disturbances may be analyzed separately. As for animal studies, we should pay more attention to the different regions (the striatum, the hypothalamus, etc.) and species differences when we compare the various conclusions from a bulk of published work. For example, anatomical studies and physiological experiments have suggested significant interspecies differences in the distribution of the cannabinoid 1 receptor both in central and peripheral nervous system (Howlett et al., 2002). Furthermore, since some genes participate in the metabolic side effects as well as the pharmaceutical effect, the functions of the AAPDs can be complex to analyze.

Genetic correlation study consists of single nucleotide polymorphisms, haplotype analysis, gene-gene interaction, genome wide association study (GWAS), etc. Several susceptibility gene loci have been reported but the exact mechanism hasn’t been illuminated. Additionally, correlations do not imply causations. Hence, *in vitro* and *in vivo* studies are needed to explore the specific effects of AAPDs on these related genes and gene-gene interactions. Further functional analyses are also required to verify which are the functional polymorphisms and the specific functional consequences of these SNPs. Moreover, both obesity and schizophrenia are polygenic diseases, so we can speculate that the metabolic side effects of atypical antipsychotics cannot be monogenic. Research strategies of monogenic diseases are inapplicable to find out the complex causations currently. Given the limitations of such research, more efficient methods are in great need. The International Schizophrenia Consortium proposed a polygenic risk score (PRS) test for schizophrenia (International Schizophrenia C. et al., 2009). Now schizophrenia polygenic risk score has been reported to be a potential predictor of antipsychotic efficacy in patients with first-episode psychosis (Jian-Ping Zhang et al., 2018). In addition, the hypothesis of an omnigenic model proposed by Boyle et al. provide us with a new perspective to understand gene effects—core genes and peripheral genes. (Boyle et al., 2017).

## Future Perspective

Although the pharmacological mechanisms and pharmacogenomics of atypical antipsychotic drugs seem to be hard to figure out, research on weight gain and dyslipidemia induced by atypical antipsychotics is of great significance. On one hand, combining genetic markers and relevant clinical indicators, a pharmacogenetic model can be built to predict the risk of lipid disturbances for an individual patient using atypical antipsychotics. Consequently, it will allow clinicians to select appropriate medication with less metabolic side effects and enough efficacy for every patient and promote individualized treatment. A multigene risk-model has showed promising results of predicting antipsychotic-induced weight gain (Tiwari et al., 2014). Moreover, the combinatorial model of genetic and clinical data could help to identify patients at high risk for early weight gain (Vandenberghe et al., 2016). On the other hand, the more we know about the mechanism of the metabolic side effects of atypical antipsychotics, the better we can tackle with this troublesome adverse drug reaction. More specifically, maybe we can develop novel molecules with high receptor selectivity or new drug targets. And toxicities could be designed out by counter-screening approaches combined with medicinal chemistry methods when discovering selectively non-selective drugs representing highly effective treatments (Roth et al., 2004). Besides, combination with lipid-lowering medication might help to attenuate weight gain or dyslipidemia. Metformin is a good try. In a double-blind and placebo-controlled study, metformin addition showed great efficacy, safety, and good adherence in preventing olanzapine-induced weight gain in drug-naive first-episode schizophrenia patients (Wu et al., 2008). In addition, recently a preclinical study indicates that the selective protein kinase C*β* (PKC*β*) inhibitor, ruboxistaurin (LY-333531) prevent long-term clozapine-induced weight gain through the inhibition of the lipid droplet-selective autophagy process (Rimessi et al., 2017). In summary, further studies focusing on the prediction model and drug combination are needed. Decreasing the adverse drug reaction will help improve the compliance of patients, ensure the therapeutic effect, and promote the life quality of them.

## Author Contributions

NL wrote the manuscript and designed the figures and the table. TC and XW participated in the survey of the literatures and organization of the table. MT and DX contributed to manuscript reviewing and revisions. HC conceived the idea, supervised the whole work, and critically revised the paper.

## Funding

This work was supported by the National Natural Science Foundation of China (81401113) and the Natural Science Foundation of Hunan Province (2017JJ3444).

## Conflict of Interest

The authors declare that the research was conducted in the absence of any commercial or financial relationships that could be construed as a potential conflict of interest.

## Abbreviations

AAPD, atypical antipsychotic drug; 5-HT, 5-hydroxytryptamine/serotonin; DRD2, dopamine receptor D2; LEP, leptin; NPY, neuropeptide Y; MC4R, the melanocortin 4 receptor; BDNF, the brain-derived neurotrophic factor; CNR1, the cannabinoid 1 receptor; *INSIG*, insulin-induced gene; ADRA2A, adrenergic α-2A receptors; SNP, single nucleotide polymorphism; BMI, body mass index; HDL, high-density lipoprotein; AgRP, agouti-related protein; CART, amphetamine-regulated transcript; POMC, pro-opiomelanocortin.
